# SOD1 Overexpression Preserves Baroreflex Control of Heart Rate with an Increase of Aortic Depressor Nerve Function

**DOI:** 10.1155/2016/3686829

**Published:** 2015-12-28

**Authors:** Jeffrey Hatcher, He Gu, Zixi (Jack) Cheng

**Affiliations:** ^1^Biomolecular Science Center, Burnett School of Biomedical Sciences, College of Medicine, University of Central Florida, Orlando, FL 32816, USA; ^2^Department of Anesthesia, University of Iowa Hospital and Clinics, Iowa City, IA 52242, USA

## Abstract

Overproduction of reactive oxygen species (ROS), such as the superoxide radical (O_2_^∙−^), is associated with diseases which compromise cardiac autonomic function. Overexpression of SOD1 may offer protection against ROS damage to the cardiac autonomic nervous system, but reductions of O_2_^∙−^ may interfere with normal cellular functions. We have selected the C57B6SJL-Tg (SOD1)2 Gur/J mouse as a model to determine whether SOD1 overexpression alters cardiac autonomic function, as measured by baroreflex sensitivity (BRS) and aortic depressor nerve (ADN) recordings, as well as evaluation of baseline heart rate (HR) and mean arterial pressure (MAP). Under isoflurane anesthesia, C57 wild-type and SOD1 mice were catheterized with an arterial pressure transducer and measurements of HR and MAP were taken. After establishing a baseline, hypotension and hypertension were induced by injection of sodium nitroprusside (SNP) and phenylephrine (PE), respectively, and ΔHR versus ΔMAP were recorded as a measure of baroreflex sensitivity (BRS). SNP and PE treatment were administered sequentially after a recovery period to measure arterial baroreceptor activation by recording aortic depressor nerve activity. Our findings show that overexpression of SOD1 in C57B6SJL-Tg (SOD1)2 Gur/J mouse preserved the normal HR, MAP, and BRS but enhanced aortic depressor nerve function.

## 1. Introduction

Autonomic control of the cardiovascular system is compromised in multiple disease conditions such as diabetes [1–3], hypertension [4], sleep apnea [5], and aging [6–9]. One of the autonomic functions impacted is the baroreflex control of heart rate (HR). Baroreflex sensitivity (BRS) is a measure of the strength of baroreflex control of heart rate in response to changes in arterial pressure, and it is an important index of cardiac autonomic function. Several clinical conditions are strongly associated with an attenuated BRS, including hypertension [10], vasovagal syncope [11], and heart failure [12], and it is considered as an independent risk factor for cardiac failure and sudden death [13–15].

The baroreflex arc is composed of multiple neural components including the aortic and carotid baroreceptors, the nucleus tractus solitarius (NTS), paraventricular nucleus of the hypothalamus (PVN), nucleus ambiguus (NA), caudal ventrolateral medulla (CVLM), and rostral ventrolateral medulla (RVLM) [16–19]. An increase in levels of reactive oxygen species (ROS) in these neural components of the baroreflex arc including the carotid and aortic baroreceptors [20–23], NTS [24–26], PVN [27], and RVLM [28–30] are seen in conditions such as diabetes, sleep apnea, hypertension, and heart failure.

Several lines of research suggest that antioxidant therapy, whether applied locally [20, 21, 31] or systemically [32–34], can restore normal baroreflex function in some of these disease states, ostensibly by decreasing levels of ROS species. However, ROS, including the superoxide radical, play critical roles in regulating the firing properties of neurons [35]. For instance, the ANGII signaling pathway is dependent upon superoxide anions generated by the actions of NADPH oxidase [24, 36]. Indeed, some studies have noted that superoxide scavenging can affect central regulation of heart rate and blood pressure in healthy animals as well as some disease models [31, 32, 37].

Conversely, other investigations have suggested SOD1 overexpression can be detrimental to neuronal tissues. Some studies [38, 39] found evidence of mild axonal degeneration and some death of motor neurons in mice overexpressing hSOD1. In addition, increased lipid peroxidation [40, 41], increased sensitivity to kainic acid excitotoxicity [42], and impaired recovery following nerve injury [43] have been reported in hSOD1 transgenic mice. Though SOD1 overexpression is protective up to a certain level, further increases in expression may contribute to peroxide formation and deleterious sequela for the tissues [44, 45]. SOD1 overexpression has also been investigated as a contributor to the pathology of Down syndrome, a condition in which SOD1 overexpression is well documented [46–48].

All together, the available evidence from the literature has suggested that SOD1 overexpression can have* either* protective* or* detrimental effects on tissues. Therefore, we need to consider if hSOD1 overexpression in animals can affect the neural components of the baroreflex loop in healthy animals. Only then, we can consider any potential benefits of SOD1 overexpression in chronic intermittent hypoxia-, diabetes-, and aging-induced impairment of baroreflex arc as shown in our previous studies [49–57]. In the present study, we have determined the effects of hSOD1 overexpression in transgenic mice on several physiological variables [arterial pressure (AP), heart rate (HR), baroreflex sensitivity (BRS), and aortic depressor nerve function] compared to controls. Our data indicated that hSOD1 overexpression in transgenic mice did not alter the values of AP, HR, and baroreflex sensitivity but enhanced aortic depressor nerve function. This study will provide baseline data on the hSOD1 overexpressing mouse line in order to facilitate future studies on possible baroreflex protective effects of overexpressed SOD1 in murine disease models.

## 2. Materials and Methods

### 2.1. Animals

Mice (C57BL/6j 3-4 mo, *n* = 16) were used as controls for the transgenic human Cu/Zn SOD overexpressing mice (C57B6SJL-Tg (SOD1)2 Gur/J, Jackson catalog # 002297, *n* = 16). Procedures were approved by the University of Central Florida Animal Care and Use Committee and followed the guidelines established by the National Institutes of Health. Efforts were made to conduct the experiments humanely and to minimize the numbers of animals used.

#### 2.1.1. Surgical Procedure

The surgical procedure has been described previously in detail [52]. Briefly, mice were anesthetized with 3% isoflurane inhalation and maintained with 1% in a 95% O_2_ and 5% CO_2_ through a tracheal tube which was connected to a rodent ventilator, as in our previous studies [51–53].

Depth of anesthesia was carefully monitored by eye blink, withdrawal reflexes (toe and tail pinch), and fluctuations of AP. Body temperature was maintained at 37 ± 1°C with a homeostatic plate and a rectal probe (ATC 1000; World Precision Instrument, Sarasota, FL, USA). The tips of plastic catheters (polyethylene-50) were tapered to ~0.3 mm diameter, the right femoral artery and left femoral vein were exposed, and the tapered ends of the two catheters were filled with heparinized saline and inserted into the femoral artery and vein. Measurement of AP was through the artery. Vasoactive drugs sodium nitroprusside (SNP) and phenylephrine (PE) were infused into the femoral vein using a microinfusion pump. These mice were used for AP, HR, baroreflex bradycardia and tachycardia, and aortic depressor nerve (ADN) recordings.

#### 2.1.2. Baroreflex Sensitivity

**Figure 1:**
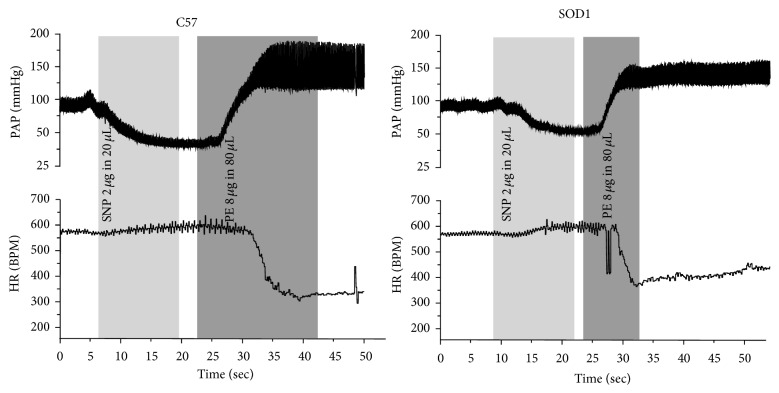
Original recordings of heart rate responses to pulse arterial pressure changes (PAP) induced by the sequential administration of SNP/PE in representative C57 and SOD1 animals. A tachycardiac phase was induced by a baroreflex-mediated increase in heart rate in response to decreased blood pressure due to SNP infusion. A bradycardic phase was induced by a baroreflex-mediated decrease in heart rate in response to increased blood pressure caused by injection of PE. During SNP application, MAP and HR changes from the baseline of MAP to the nadir of MAP were measured as shown in the light gray box. During PE application, MAP and HR changes were measured from the peak of the HR to the nadir of the HR as shown in the dark gray box.

The blood pressure catheter was connected to a pressure transducer (MIT0699, AD instruments) which was connected to the PowerLab Data Acquisition System (PowerLab/8 SP). Baseline values of mean arterial pressure (MAP) and HR and the MAP and chronotropic responses to sequential SNP/PE applications were measured using Chart 5 software (AD instruments). SNP and PE (Sigma, St. Louis, MO, USA) were freshly prepared, diluted in 0.9% NaCl, and infused by sequential bolus injections. SNP [2.3 ± 0.1 *μ*g (C57) versus 2.2 ± 0.1 *μ*g (SOD1) in 0.1 *μ*g/*μ*L saline, *P* > 0.05] was first injected, and after 10–20 s PE [8.1 ± 0.1 *μ*g (C57) versus 6.9 ± 0.4 *μ*g (SOD1) in 1 *μ*g/*μ*L saline, *P* < 0.05] was then injected. Such doses of sequential bolus injections of SNP/PE could induce a fast and large decrease followed by an increase of the AP. The measurement of decrease and increase in the AP was completed in ~1 min to reduce possible baroreflex resetting. Using these doses, MAP decreases and increases were quite comparable in control and SOD1 mice. Baseline values of MAP and HR were measured from a 30 s interval before SNP injection. After injections, MAP and HR returned to baseline values normally within 10–20 min. HR responses to MAP changes induced by sequential administration of SNP and PE included two phases: tachycardiac and bradycardic responses. During the tachycardiac (SNP) phase, MAP and HR changes were measured in the time window as indicated by a light gray box in Figure 1, that is, the baseline of MAP to the nadir of MAP. During the bradycardic (PE) phase, MAP and HR changes were measured in the time window as indicated by a dark gray box in Figure 1, that is, from the peak of the HR to the nadir of the HR. The MAP and corresponding HR were sampled and averaged every second. We applied linear regression analysis of the ΔHR-ΔMAP relationship for each animal, and the slope of the regression line was used as an indicator for baroreflex sensitivity (BRS) as previously described [52]. Data obtained for SNP and PE injections were averaged separately and reconstructed as separated regression lines for each group.

### 2.2. Baroreceptor Afferent Function

The aortic depressor nerve (ADN) on the left side was identified in the cervical region using a dissecting microscope. The left ADN was carefully isolated from surrounding connective tissues with fine glass tools to avoid stretching or injury of nerve. After that, the left ADN was placed on miniaturized bipolar platinum electrodes (outer diameter: 0.12-mm). The nerve and electrodes were soaked in mineral oil. Aortic depressor nerve activity (ADNA) was amplified (×10,000) with the band-pass filters set between 300 and 1000 Hz by an AC Amplifier (Model 1800, A-M Systems, Sequim, WA, USA). The ADNA, integrated ADNA, phasic arterial pressure (PAP), HR, ECG, and body temperature were all recorded and simultaneously displayed on different channels of the PowerLab System. Chart 5.2 software and Sigma Plot 9.0 were used for data acquisition, analysis, and presentation. All ADNA for analysis had a signal-to-noise ratio > 10 : 1.

The ADNA signal occurred as rhythmic bursts that exhibited cardiac cycles and were synchronized with PAP (Figure 3). ADNA signal was integrated using a 10 ms time constant to obtain the integrated ADNA (Int. ADNA). The “ADNA silent” or the “noise level” between the ADNA bursts was averaged from 30 “ADNA silent” intervals and was used to determine the noise level for Int. ADNA as shown in Figure 3. This averaged noise level was subtracted from original Int. ADNA signal to obtain the corrected Int. ADNA with the averaged noise level of 0 *μ*V· s. The corrected Int. ADNA and MAP were used to construct baroreceptor afferent function curves using logistic sigmodal function. For simplicity, we used Int. ADNA for corrected Int. ADNA in the text below. The baroreceptor function curve was calculated at the rising phase of PE-induced AP increase starting from the nadir of the SNP-induced fall in AP to the maximum of the AP increase. R waves of ECG signal were used to automatically define cardiac cycles by the Chart 5.2 Macro function (arrows in Figure 3). The baroreceptor function curve was fitted by plotting the percent (%) of change of the mean Int. ADNA per cardiac cycle relative to the Int. ADNA baseline value before drug administration against MAP using a sigmoid logistic function [58]. The logistic function for Int. ADNA used the mathematical expression: *Y* = −*P*_1_/{1 + exp⁡[*P*_2_(*X* − *P*_3_)]} + *P*_4_, where *X* = MAP, *Y* = Int. ADNA (% baseline), *P*_1_ = maximum − minimum (range), Int. ADNA (range), *P*_2_ = slope coefficient, *P*_3_ = MAP at 50% of the Int. ADNA range (*P*_mid_), and *P*_4_ = maximum Int. ADNA.

The *P*_th_ and *P*_sat_ were calculated from the 3rd derivative of the logistic function, and they were expressed as *P*_th_ = *P*_3_ − (1.317/*P*_2_) and *P*_sat_ = *P*_3_ + (1.317/*P*_2_). The maximum slope or gain (*G*_max_) was calculated at *P*_mid_ from the 1st derivative of the logistic function: *G*_max_ = *P*_1_ × *P*_2_/4. Approximately 200–500 data points measured over 30–50 s were used to construct a baroreceptor function curve using Sigma Plot software. The squared correlation coefficient *R* ^2^ was used to determine the goodness of curve fitting.

### 2.3. Statistical Analysis

Data were presented as means ± S.E. Student's *t*-test was used to compare the difference of HR, MAP, slopes of the regression lines, and parameters of the baroreceptor function curves between groups. Differences were considered significant at *P* < 0.05.

## 3. Results

### 3.1. MAP, HR, and SNP/PE-Induced MAP Changes

hSOD1 overexpression did not alter baseline MAP and HR, and SNP-induced minimums and PE-induced maximums for MAP changes in C57 and SOD1 mice were comparable (Table 1), which allowed us to investigate baroreflex control of HR over a similar range of blood pressure changes (see the following).

### 3.2. Baroreflex Sensitivity

**Figure 2:**
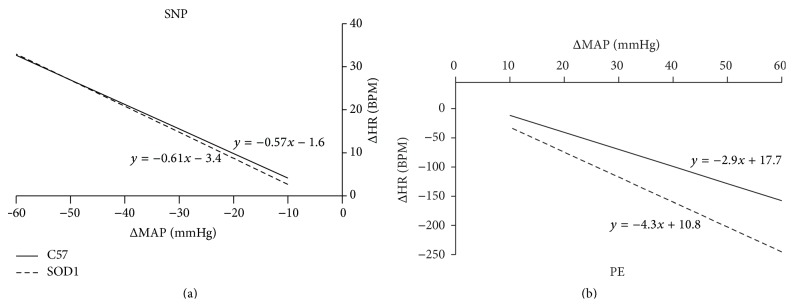
Baroreflex sensitivity. (a) The averaged regression lines for ΔHR/ΔMAP for the SNP-induced tachycardiac baroreflex for C57 (*n* = 8) and SOD1 (*n* = 8). Regression lines for tachycardiac baroreflex response are similar between the two groups. (b) The averaged regression lines for ΔHR/ΔMAP for the PE-induced bradycardic baroreflex for C57 (*n* = 8) and SOD1 (*n* = 8). Though visibly distinct, the slope of the regression line for SOD mice was not significantly different than that of C57 (*P* > 0.5).

The original recordings of HR changes (ΔHR) in response to sequential injections of SNP and PE were shown in Figure 1. SNP infusion (light gray box) decreased AP which induced a HR increase (tachycardiac phase). PE infusion (dark gray box) resulted in an increase in AP that drove a baroreflex-mediated reduction in heart rate (bradycardic phase). Tachycardiac and bradycardic responses (ΔHR) against ΔMAP were fitted using separate regression lines for both C57 and SOD1 animals (*n* = 8/group), respectively. The slopes of the regression lines represent the baroreflex sensitivity (BRS) and they were similar (Figures 2(a) and 2(b)) for the tachycardiac phase [C57: −0.57 ± 0.06 bpm/mmHg, SOD1: −0.61 ± 0.08; *P* > 0.05] as well as the bradycardic phase [C57: −2.9 ± 0.57 bpm/mmHg, SOD1: −4.3 ± 0.84 bpm/mmHg; *P* > 0.05]. Even though there was a trend of increased BRS for SOD1 animals during the bradycardic phase, the slopes of the regression lines were not significantly different. Therefore, overexpression of hSOD1 did not significantly change baroreflex-mediated tachycardia and bradycardia.

### 3.3. Aortic Depressor Nerve Function

**Figure 3:**
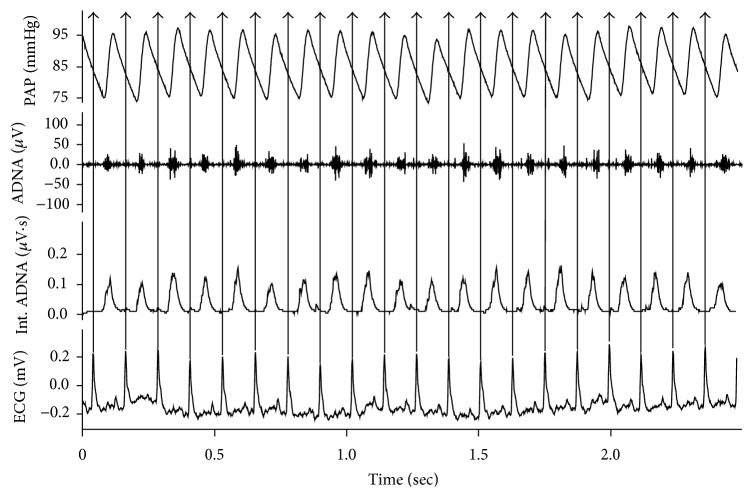
Original recording of PAP, ADNA (Raw ADNA), integrated ADNA (Int. ADNA), and ECG in aC57 mouse. Trace 1: PAP. Trace 2: ADNA occurred as rhythmic bursts that exhibited cardiac rhythmic patterns and was synchronous with PAP. Note: ADNA increased prior to AP increases. This is because the catheter for blood pressure measurement was inserted into the femoral artery. Trace 3: ADNA signal was integrated using a 10 ms time constant to obtain the Int. ADNA curve. The small boxes in the Int. ADNA trace enclose the intervals between ADNA bursts where signal noise can be measured. Trace 4: ECG. The R waves of the ECG signal were used to separate ADNA firing intervals automatically by Chart 5.2 (arrows).

**Figure 4:**
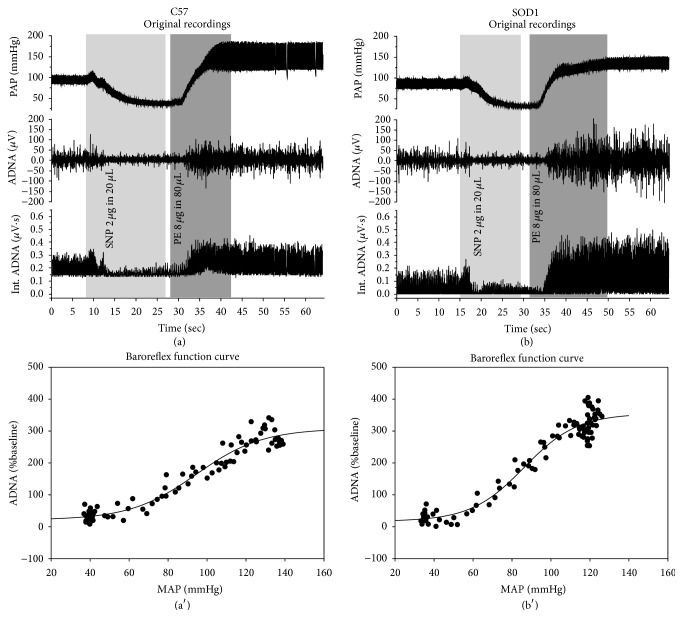
Baroreceptor function curve. (a and b) Original recordings showing pulse arterial pressure (PAP) and ADNA responses to sequential i.v. injections of SNP/PE in two representative C57 and SOD1 mice, respectively. (a′ and b′) Int. ADNA and MAP relationship curves of these two representative mice were fitted using logistic function, respectively (Int. ADNA: integrated ADNA).

**Figure 5:**
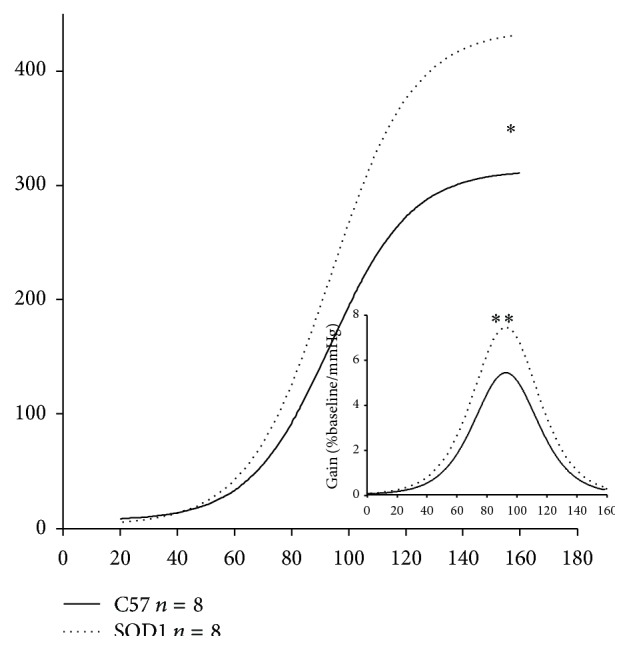
Baroreceptor afferent function: baroreceptor function curves for C57 (*n* = 8) and SOD1 (*n* = 8) groups were reconstructed using the averaged parameters of sigmoid logistic function curves (see Table 2). The baroreceptor discharge function curve for SOD1 mice is significantly higher for SOD mice compared to C57 (^*∗*^ *P* < 0.05) and the gain of the baroreceptor afferent function curve was significantly higher for the SOD1 mice than for the C57 mice (^*∗∗*^ *P* < 0.01).

Aortic depressor nerve function was measured as the aortic depressor nerve activity (ADNA) decreases in response to SNP/PE-induced AP elevation. Figure 3 shows the original recordings of typical burst ADNA in synchrony with PAP. Figures 4(a) and 4(b) show the original recordings of ADNA in response to changes in blood pressure in representative C57 and SOD1 mice. SNP injection decreased and PE injection increased ADNA. Int. ADNA and MAP relationship curves were fitted using the logistic sigmodal function in these two mice as shown in Figures 4(a′) and 4(b′). The averaged parameters of the logistic function curves (Table 2) show that the SOD1 animals had a significantly larger maximal Int. ADNA response (*P*_4_) compared to C57 (*P* < 0.05). The maximal gain of the ADNA response (*G*_max_) was also significantly greater in SOD1 than C57 (*P* < 0.01), indicating that hSOD1 overexpression resulted in more sensitive responses than the control, thus increasing the aortic baroreceptor depressor nerve function. The plots of the sigmoid logistic function curves of the averaged Int. ADNA-MAP relationship for C57 and SOD1 mice were shown in Figure 5.

## 4. Discussion

In this study, we demonstrated that the overexpression of human SOD1 in mice does not have significant effect on AP, HR, or SNP/PE-induced changes of MAP and BRS as compared to controls. However, SOD1 overexpression enhanced baroreceptor depressor nerve function in response to AP elevation. While we could not fully interpret the mechanism, Li et al. [31] measured a similar increase in baroreceptor sensitivity after application of SOD1 and catalase to the nodose ganglia of healthy rabbits, though the effect was mild and not reversed by washout of the SOD or catalase.

### 4.1. hSOD1 Overexpression Did Not Change Basal Blood Pressure and Heart Rate

Superoxide radicals have profound effects in the modulation of neural activity in the brain stem [24, 59], and it is known that increases in ROS alter autonomic regulation of blood pressure [24, 60, 61]. One of the concerns of using the SOD1 mouse line is that interference with superoxide-dependent signaling in the brainstem by hSOD1 overexpression would alter basal HR and MAP. It is fairly well established that treatments with antioxidants may lead to changes of autonomic regulation in normal and disease models. hSOD1 overexpression in the paraventricular nucleus was found to reduce sympathetic activity and attenuate hypertension in spontaneously hypertensive rats, although no effect was detectable in the Wistar controls [27]. Another study found that endothelial-specific catalase overexpression caused a significant reduction in blood pressure in healthy mice [62]. Systemic administration of tempol, a SOD mimic [63], has previously been found to reduce MAP, HR, and renal sympathetic nerve activity (RSNA) in both normal and baroreceptor denervated rats [34]. The same study noted reduced spontaneous discharge rate of neurons in the paraventricular nucleus of the hypothalamus (PVN) and the rostral ventrolateral medulla (RVLM), two critical nuclei involved in sympathetic regulation of the cardiovascular system. A similar study performed on normotensive WKY and spontaneously hypertensive (SHR) rats also demonstrated reduced HR and MAP during systemic tempol administration in both groups of animals, as well as decreased splanchnic nerve activity [32]. Kawada's study determined that the reduction in blood pressure was not caused by changes in peripheral vascular tone in WKY animals, although AP reduction was associated with relaxation of vascular tone in the SHR rats. Taken together, these prior studies raise the possibility that superoxide radical scavenging may have effects on neural and vascular control of blood pressure in diseased and healthy animals.

In our study, hSOD1 overexpression had no significant effect on MAP, HR, and BRS in healthy mice. This is in agreement with a previously published report [64] showing blood pressure equivalence between C57bl/6J mice and 6-TgN(SOD1)3Cje mice with a 3-fold overexpression of hSOD1 (Jackson Catalog#: 002629). Therefore, hSOD1 overexpression in healthy animals does not seem to compromise basic hemodynamic stability as measured by HR and AP.

### 4.2. Baroreflex Control of Heart Rate Not Significantly Affected by hSOD1 Overexpression

Previous studies have shown that redox species modulate the activity of baroreceptor neurons [20–23], NTS [24–26], sympathetic brain stem nuclei such as the PVN [27] and RVLM [28–30], and intrinsic cardiac ganglia [65]. Perturbations in the function of any of the components of the baroreflex loop can alter baroreflex sensitivity and function. Indeed, it is well established that increased ROS levels in these components can reduce baroreflex sensitivity and response [26, 31, 61]. However, the effect of long-term antioxidant supplementation, and in particular SOD1 overexpression, on baroreflex function in healthy animals was less well documented. Li et al. [31] found that exogenous SOD1 or catalase applied to the carotid sinus caused a small but significant increase in baroreceptor activity between the pressures of 60 and 80 mmHg but did not increase the maximal baroreceptor activation. However, this study did not measure HR response to blood pressure ramps (ΔHR/ΔMAP), so it is uncertain if local application of SOD or catalase to the carotid baroreceptors would have had an appreciable effect on the baroreflex control of heart rate. Guimarães et al. [66] investigated the effects of NADPH-derived superoxide anion reductions by IV Tiron (a superoxide anion scavenger, [67]) or apocynin (NADPH oxidase inhibitor, [68]) on BRS in WKY and SHR rats and found that the acute application of these agents had no significant effect on BRS in healthy animals, although they improved BRS in the hypertensive animals. Unfortunately, this still does not give any indication as to what effects long-term superoxide anion scavenging could have on BRS. The results of our investigation suggest for the first time that moderate (~3.5-fold, [69]) chronic systemic hSOD1 overexpression does not significantly impact baroreflex sensitivity in healthy animals.

### 4.3. Baroreceptor Afferent Function Enhanced by Overexpression of Human SOD1

As shown in Figure 5, we found that hSOD1 overexpressing mice showed significantly increased aortic baroreceptor activation slope and gain compared to C57 controls. This result is similar to the finding of Li et al. [31], who reported that carotid sinus nerve (CSN) activation in response to BP ramps was enhanced by application of SOD or catalase to the carotid sinus in rabbits.

Defining the mechanism the observed increase in baroreceptor activation is beyond the scope of this investigation, but there are certainly precedents in the literature. SOD1 is known to support nitrous oxide (NO) mediated vasorelaxation [70], which improves arterial compliance [71–73]. A high degree of vascular compliance is linked to robust baroreflex sensitivity [9, 74, 75], which decreases in step with vascular compliance. There is research that supports the hypothesis that antioxidant supplementation can improve large artery compliance [76, 77].

SOD1 may directly affect the mechanosensory properties of the aortic baroreceptor terminals by altering the expression or activity of critical ion channels in the baroreceptor terminals. There is evidence that ROS inhibit the expression of ASIC2 (Acid-Sensitive Ion Channel) which is critical to mechanotransduction of arterial pressure [78, 79]. ASIC2 expression is downregulated in SHR rats [80, 81], which show a similar diminution of baroreceptor activation to ASIC2 null mice [79]. Application of the superoxide mimetic tempol to nodose ganglia cells in either the ASIC2 null mice or SHR rats has been shown to restore baroreflex activation of baroreceptor neurons to near normal levels [78]. Hyperpolarization-activated cyclic nucleotide (HCN) channels, which are also strongly linked to mechanoreception in arterial baroreceptors, are shown to be upregulated in type 1 diabetes mellitus leading to a reduced baroreceptor function that can be rescued with tempol [22].

### 4.4. Perspectives

A growing body of evidence has shown that interventions based on antioxidants can be effective in reducing hypertension, increasing vascular compliance and function, and improving baroreflex-mediated control of heart rate [25, 27, 32, 33, 66, 76]. However, because ROS have a wide variety of necessary biological functions, it is important to evaluate the effect of systemic application of antioxidants on cardiac autonomic function in the absence of any other diseases. The current study uses a transgenic mouse model based on the C57bl/6j mouse line which has been engineered to express human Cu/ZnSOD (SOD1) at a level roughly 3.5-fold over normal murine SOD1 expression in cortical tissue [69]. This transgenic mouse line was originally developed in 1994 as a gene-dosage control for a mouse model of amyotrophic lateral sclerosis (ALS) overexpressing a SOD1-G93A mutation. The SOD1 mouse line used in this study showed no signs of ALS-like symptoms. In addition, Dal Canto and Gurney [39] also performed anatomical assessment of neural tissue and found that the mice overexpressing hSOD1 showed very subtle changes in the anterior portion of the anterior horn (mild swelling of motor fibers and vacuolization of dendrites) but were free of any ALS-like symptoms [39]. Functionally, there were no signs of impaired motor performance until 58 weeks of age. tgSOD1 mice in the current experiment were between 12 and 16 weeks of age, well before the window in which motor function changes are observed. In our study, we did not find any changes in AP, HR, vasoactive drugs-induced hypo- and hypertension, and BRS. Noticeably, the aortic depressor nerve function is increased in hSOD1 mice. Even though we could not interpret the mechanism for such an enhancement of aortic depressor nerve function, it appears that hSOD1 overexpression in this line of mouse did not impair but may have increased the function of baroreceptor afferent components in the baroreflex arc. Interestingly, Xu et al. [82] reported that mice overexpressing hSOD1, the same model as the one we used in present study, showed increased resistance to oxidative stress and apoptosis of cortical neurons after exposure to chronic intermittent hypoxia compared to wild-type control. hSOD1 overexpression has also been shown to protect against mitochondrial cytochrome C release and subsequent apoptosis in focal cerebral ischemia models of stroke [83]. Since our data indicate that hSOD1 overexpression did not cause dysfunction of MAP, HR, and BRS but may increase aortic baroreceptor nerve function, we suggest that this model can be potentially used to study whether increased expression of hSOD1 protects against disease (such as chronic intermittent hypoxia and diabetes-) induced impairment of baroreflex sensitivity, vagal motor neuron death in the nucleus ambiguus, and degeneration of vagal afferent and efferent axons in the aortic arch and cardiac ganglion shown in the previous studies [51, 52, 55, 84–86]. Whether the increased baroreceptor sensitivity in healthy animals may prove advantageous in mitigating disease-induced impairment of autonomic control of the heart is a promising concept, and we are currently using our hSOD1 overexpressing mice to determine if hSOD1 overexpression can preserve normal afferent, efferent, and central components of the baroreflex arc in the CIH model of sleep apnea.

It should be pointed out that since baroreflex-mediated reduction of heart rate in response to increased arterial pressure had a trend of increase but not significantly, it appears that the increased signaling from the aortic depressor baroreceptor nerves to the brainstem is buffered by other neural components of the baroreflex loop, such as the NTS, NA, or cardiac ganglia within the heart. Whether such a buffering is a product of normal physiologic compensation or something of a more pathologic nature is undetermined. Thus, careful studies of other neural components in the baroreflex arc in addition to aortic depressor nerves are critically important to fully understand the effects of hSOD overexpression on the whole baroreflex circuitry.

## Figures and Tables

**Table 1:** There were no significant differences in baseline heart rate (HR) and mean arterial pressure (MAP) between C57 and SOD1 mice. Depression in arterial pressure following exposure to SNP, or increase in arterial pressure after PE infusion, was also similar between the two groups of animals.

Animal group	Average HR (BPM)	MAP (mmHg)	SNP MAP (mmHg)	PE MAP (mmHg)
C57 (*n* = 8)	558 ± 8	88.8 ± 2.9	38.7 ± 1.4	135.8 ± 3.1
SOD1 (*n* = 8)	553 ± 13	85.8 ± 2.1	39.5 ± 1.3	136.6 ± 3.5

**Table 2:** Parameters defining the baroreceptor afferent function curve (ADNA% baseline) in C57 and SOD1 mice.

	*R* ^2^	*P*_1_ (%)	*P*_2_	*P*_3_ (mmHg)	*P*_4_ (%)	*G*_max⁡_ (%/mmHg)	*P*_th_ (mmHg)	*P*_sat⁡_ (mmHg)
C57 (*n* = 8)	0.95 ± 0.01	−307 ± 19	0.07 ± 0.006	94 ± 3	314 ± 18	5.4 ± 0.3	74 ± 3	114 ± 4
SOD1 (*n* = 8)	0.95 ± 0.01	−434 ± 44	0.07 ± 0.004	94 ± 3	436 ± 37	7.4 ± 0.5	73 ± 2	114 ± 4
*P* value	N.S	*P* < 0.02	N.S	N.S	*P* < 0.02	*P* < 0.01	N.S	N.S
